# Educational Services and School Reintegration Supports for Youth After Acute Behavioral Health Unit Hospitalization

**DOI:** 10.5334/cie.178

**Published:** 2025-10-29

**Authors:** Heather Ormiston, Polly Husmann, Kristin Wikel, Debra Reisinger, Michelle Curtin

**Affiliations:** 1 Indiana University, US; 2 Indiana University School of Medicine, US; 3 St. Vincent’s Peyton Manning Children’s Hospital, US; 4 Cincinnati Children’s Hospital Medical Center, US; 5 Wake Forest University School of Medicine, US

**Keywords:** school reintegration, inpatient hospitalization, behavioral health, educational services

## Abstract

Youth with mental health needs significant enough to warrant inpatient psychiatric hospitalization are on the rise. After inpatient hospitalization, youth transitioning back to their school of record need continued support to promote recovery. Little empirical work has been conducted to understand the educational experiences of youth hospitalized for mental health needs. Further, there is a need to document the services provided to youth upon discharge and return to their school of record. Thus, the purpose of this empirical study is to better understand the patient population (n = 264, mean age = 14.04 years old; 70.1% White, 73.2% female) of an acute behavioral health unit in a free-standing Midwestern children’s hospital and the educational services (e.g., consultation, direct instruction, support for reentry, and educational advocacy services) provided by the Hospital-Based School program prior to, during, and post-hospitalization to those students. Significant differences in educational services were identified based on some student demographic characteristics such that older students were more likely to have a 504 plan at admission, and individuals with neurodevelopmental disorders, psychosis, and other mental disorders were found to be most likely to have special education services prior to admission. Implications for practice related to improved care coordination upon reentry between hospitals and schools and the role of schools in the provision of behavioral and mental health services, limitations of the current study, and directions for future research are discussed.

Youth with behavioral health needs encompass individuals with “mental, behavioral, emotional, developmental, and substance use disorders” (Choi & Easterlin, 2018, p. 754). Youth returning to school after an acute behavioral health (ABH) unit hospitalization are often faced with the stressful transition of having to reintegrate into the educational environment (Blizzard et al., 2016; Marraccini et al., 2019). Youth and their families have expressed concerns with managing relationships with peers and school personnel upon their return, missing academic content and being academically behind, and perceived embarrassment and stigma associated with navigating the social and educational landscape following ABH unit hospitalization (Blizzard et al., 2016; Ogilvie et al., 2019; Preyde et al., 2017). The acute and significant socioemotional, academic, and cognitive needs of youth upon discharge (Marraccini et al., 2019) often require significant support upon reintegration such as adjustments to special education programming and classroom placement (Midura et al., 2023), particularly as families advocate for these types of supports (Blizzard et al., 2016; Midura et al., 2023; Tougas et al., 2019). Yet, youth making the transition back to school often lack school-based supports needed to “maintain gains achieved during hospitalization” (Midura et al., 2023, p. 24). As demonstrated in the extant literature, when youths’ mental health needs are not prioritized during the posthospitalization and reintegration period, youth are at significant risk of rehospitalization (Midura et al., 2023). However, the successful reintegration of youth into the school setting upon discharge from ABH is a critical element to promote recovery post-hospitalization (Tougas et al., 2019), in part to improve services provided by schools to support the returning youth (Marraccini et al., 2019). In consideration of the International Classification of Functioning, Disability and Health – Children and Youth Version (ICF-CY; World Health Organization [WHO], 2007), successful reintegration focuses on not only what youth can do in the immediate post-hospitalization period but also trajectory planning to increase their ability to fully participate in age-appropriate activities. However, a variety of factors are currently impediments to this success, for which Hospital-Based Schools Programs may be situated to support, as discussed below. Thus, this element of service delivery is important to examine to optimize the socioemotional and mental well-being of youth post-discharge.

## Defining Key Terms

For purposes of the present manuscript, it is necessary to define key terms associated with the study. Specifically, behavioral health is a construct that is used to encompass individuals’ mental health diagnoses and conditions, mental distress, and behaviors such as substance use, suicidal ideation, and suicidal behaviors (Centers for Disease Control and Prevention, 2024). As such, for the purposes of the present study, “mental health” is subsumed within “behavioral health” and we are intentional about our use of both terms throughout this manuscript particularly since the extant literature reviewed in this manuscript address both constructs in their own right. Further, in relation to the setting of the present study, youth hospitalized for both mental health concerns (e.g., depression, anxiety) as well as behavioral health concerns (e.g., suicidal ideation) are housed within the pediatric acute behavioral health (ABH) unit, although this phrase is also referenced in various capacities in the literature with terms such as psychiatric inpatient (e.g., Safer, 2021) and mental health inpatient units (e.g., Hayes et al., 2018), among others.

### Acute Behavioral Health Unit Hospitalizations

ABH hospitalization is warranted for youth with significant behavioral health needs, defined as being a danger to themselves or others (Cummings et al., 2016). Rates of hospitalization due to these significant behavioral health needs (e.g., suicidality) have increased in recent years (Arakelyan et al., 2023; Tougas et al., 2019) with an average length of stay (LOS) of six days (Safer, 2021) and typical range between five and 22 days (Olgivie et al., 2019). According to the Kids’ Inpatient Database, a national data set examining acute care discharges in the United States, youth inpatient hospitalizations for mental health needs constituted 25% of all inpatient stays in 2019, with 64% of those stays related to self-injury or suicide attempt (Arakelyan et al., 2023).

Outcomes of hospitalized youth are troubling. The time frame immediately following ABH unit hospitalization (e.g., seven to 30 days; Edgcomb et al., 2020) places youth at an increased risk of rehospitalization (Marraccini, Pittleman, et al., 2022). Estimates indicate repeat hospitalizations range between 22 and 38% in the year following an individual’s previous initial inpatient behavioral health stay (Edgcomb et al., 2020; Tougas et al., 2019). Additionally, there is a significant risk of suicide and suicide attempts for patients in the months following ABH hospitalization (Fontanella et al., 2020; Marraccini, Pittleman, et al., 2022). The increased risk for rehospitalization coupled with more intensive needs upon re-admission led researchers to question “whether hospitalized youth may not be benefiting sufficiently from treatment provided in less intensive settings” (Meagher et al., 2013, p. 167).

### Hospital-Based School Programs

Hospital-based school programs (HBSPs) play an integral role in the provision of educational services while youth are hospitalized (Steinke et al., 2016) and support transition planning once youth are discharged (Ormiston et al., 2024). HBSPs engage in direct educational service delivery such as one-on-one instruction and supporting work completion for assignments provided by the student’s home school (Boff et al., 2021). In anticipation of discharge from ABH, HBSPs often support school reentry planning via the coordination of services with youth, families, schools, and community mental health agencies (Ormiston et al., 2024; Weiss et al., 2015) to minimize the risk of readmission (Weiss et al., 2015) and explore patient and family centered values for care. Considering youths’ concerns with social dynamics and stigma related to ABH hospitalization (Marraccini et al., 2019; Marraccini, Resnikoff et al., 2022; Preyde et al., 2017), HBSPs can also provide education for educators and peers to learn about the academic and social needs of the returning individual (Ormiston et al., 2024).

## Mental Health Services for Youth

Despite a growing need, mental health services for youth are severely limited (Duong et al., 2021). For instance, less than half of rural counties across the United States have outpatient mental health facilities or behavioral health treatment providers that can provide mental health services to youth (Andrilla et al., 2018; Cummings et al., 2013). Further, utilizing data from a national sample, Cummings and colleagues (2016) found youth living in rural areas, in counties with predominantly non-White residents and counties with a high percentage of residents that are uninsured were significantly less likely to have a community-based facility that treated youth with mental health needs. When youth do access community mental health supports, the continued receipt of those services is limited—indeed, most youth receive four or fewer sessions in community mental health settings (Hoover & Bostic, 2021) when they do seek treatment. Specifically examining service use by race and ethnicity, a national study of racial and ethnic disparities in inpatient mental health care revealed White and Black youth had similar rates of psychiatric inpatient use while Latine youth had lower rates of inpatient utilization (Marrast et al., 2016). Of further concern is evidence to indicate that racial disparities related to the lack of mental health services “contribute to the overrepresentation of youth of color in the justice system, as White youth receive treatment services in the mental health system and youth of color are funneled to the juvenile justice system” (Lee et al., 2017, p. 23). Thus, while it appears rural and minoritized youth likely face multiple barriers to receipt of mental health services, ABH services, and post-discharge reintegration (Cummings et al., 2016; Marrast et al., 2016), there is limited understanding of how other demographic characteristics (e.g., sex, socioeconomic status) may play a role in service delivery and utilization post-discharge demonstrating an area in need of further research.

Schools play an important role in the provision of mental health services for youth (Ali et al., 2019) who otherwise may have limited access to such services. Indeed, schools have long been called upon to provide mental health services to youth (e.g., Weist, 1997) and recent evidence indicates schools are one of the most common providers of mental health services for youth (Duong et al., 2021). For youth with behavioral health needs, schools, and school mental health personnel more specifically, may refer youth with acute needs to facilities providing emergency services (e.g., emergency room; acute psychiatric care hospital) or may refer youth to community mental health agencies for longer-term treatment (Grant et al., 2023; Marraccini et al., 2019). Further, schools are also the primary environment in which the youth return upon ABH discharge (Marraccini, Resnikoff et al., 2022; Vanderburg et al., 2023). In this vein, re-integration support services for hospitalized youth have been found to be positively associated with the perceived quality of support services received upon reentry to school (Marraccini et al., 2019).

### Care Coordination

Despite a recommendation that reentry plans be put in place for youth upon reintegration (Marraccini, Pittleman et al., 2022), schools face significant barriers when students return from an ABH hospitalization, including a lack of health insurance coverage for school transition planning (Midura et al., 2023), a lack of training for school staff (Ormiston et al., 2024), as well as limited guidelines and structured protocols for *how* to conduct transition planning (Chen et al., 2022; Midura et al., 2023). To facilitate these transitions, care coordination has been identified as an important element to the provision of mental health services for youth with medical and behavioral health needs. Care coordination acts by connecting families with community resources to meet student needs (Francis et al., 2021; Kutash et al., 2015) including connecting youth to mental health services in the school setting (e.g., Francis et al., 2021; Nygaard, Ormiston et al., 2024). Care coordination, “defined as the deliberate organization of activities required to facilitate care amongst the breadth of individuals that may be involved in the provision of services required for an individual’s care” (Nygaard, Ormiston et al., 2024, p. 1), typically involves communication between the individual’s medical home, community providers involved in the individual’s care, and the school (McClain et al., 2020; Nygaard, Ormiston et al., 2024). For instance, care coordination for the student’s mental health needs via the provision of school mental health services can help support youth upon discharge, particularly when care is coordinated with outpatient behavioral health and medical care (e.g., medication management) needs (Francis et al., 2021).

#### Student Centered Care Coordination

In 1989, the United Nations (UN) Convention on the Rights of the Child adopted Article 12 emphasizing the active involvement of children in decisions that directly impact them and underscores that all decisions made are done so within the child’s best interest. This was further emphasized with the ICF-CY model (WHO, 2007) which contextualizes a child’s functioning within their environment, including the family context and their psychosocial functioning. In the context of care coordination and decision-making, these tenets remain at the forefront decades later. Employing strengths-based approaches are essential to this process and student engagement in care planning is an essential element to reintegration (Midura et al., 2023). Contextualized within care coordination is consideration of the child’s physical, social, and psychological development (Capurso et al., 2025; Tomberli & Ciucci, 2021). Youth sense of school belonging, for instance, is an important element for successful reintegration as connection and relatedness with the youth’s school and classmates have been linked to increase quality of life for youth with chronic illness and can help support integration, and re-integration, within the school environment (Tomberli & Ciucci, 2021). Additionally, peer-involved and peer-supported interventions (e.g., peer mentoring, peer tutoring) within the school environment have been found to reduce social isolation and increase academic performance upon reentry (Tomberli & Ciucci, 2021; Tougas et al., 2023).

### Individualized Education Plans and Section 504 Plans

Delivery of mental health services can operationalize in several formats within US schools (see Supplemental Table 1). One approach is via delivery of services through a multi-tiered system of support (MTSS) framework. Although a full review of MTSS is beyond the scope of this paper, mental health services may be delivered via the third tier within an MTSS framework, reserved for those with the most intensive level of need (Arora et al., 2019). Although schools are not meant to serve as a replacement for intensive mental health care for youth with significant mental health problems, for some students this is a necessary support. However, in some cases, the receipt of mental health services at school only takes place when included as a related service on a student’s Individualized Education Plan (IEP; Clopton et al., 2024; Nygaard et al., 2023), provided the student’s mental health needs are concurrent with an adverse impact on the student’s education (Skaar et al., 2021). For mental health services to be provided to youth via an IEP, the IEP team must determine if the student’s individualized needs, as a result of their identified disability, can be met via provision of those services (Spiel et al., 2014; Yell et al., 2018) in order to make sufficient educational progress (Santiago et al., 2014). However, mental health services are often not included in IEPs for a host of reasons including IEP teams failing to recognize the need to evaluate for mental services as a related service in an IEP (Etscheidt et al., 2024; Skaar et al., 2021; Yell et al., 2018). For youth returning to school post-ABH discharge, the provision of mental health services and/or special education services may be warranted (Savina et al., 2014) and should be considered during the student’s reentry planning process. Similarly, students may receive mental health services through a Section 504 Accommodation Plan (i.e., 504 Plan; Hoover & Bostic, 2021). For an individual to be eligible for services under a 504 Plan, a student must have a physical or mental impairment that significantly limits one, or more, major life activities (Zirkel, 2015). Of note, a student does not need to be eligible for special education in order to receive 504 Plan services (Madaus & Shaw, 2008).

## Purpose of the Present Study

There is little empirical work regarding the educational experiences of youth hospitalized for behavioral health needs (Ogilvie et al., 2019) as well as the services provided by HBSPs to this population (Steinke et al., 2016). Additionally, there is little empirical work examining the types of school-based services that are provided to youth prior to hospitalization, and what instructionally related services are offered during inpatient admission, in addition to what school- and hospital-based services are recommended or offered post-discharge. Finally, despite the identified needs of youth that transition from ABH to school (e.g., Marraccini et al., 2019), little research has documented the processes for reintegrating hospitalized youth with behavioral health related needs (Midura et al., 2023) and even less is known about behavioral health care disparities by race and ethnicity (Brent et al., 2020). Thus, the purpose of this study is to be both descriptive and exploratory in nature to better understand the patient population of an ABH unit and the educational services provided prior to, during, and post-discharge to those students. We sought to examine the following research questions:

1. What are the backgrounds (e.g., age, race/ethnicity, sex, school locale, diagnosis, special education/504 Plan status, average length of stay in unit, school type [i.e., public, private], and class type [e.g., general education, resource room, self-contained room]) of youth receiving services through a Midwest hospital-based school program on the behavioral health unit?
2. What hospital-based school program services (e.g., number of school consultations, number of direct instruction sessions, and post-discharge services) are offered to the patients on the behavioral health unit, how often are the services provided, and are there differences in number of services provided based on demographic characteristics or school/classroom type?
3. Are the services offered to the patients on the behavioral health unit different based on diagnosis?
4. Are there differences in services provided on the Behavior Health Unit, special education status, 504 Plan status, and patient primary diagnosis based on demographic characteristics?

### Study Context: Hospital-Based School Program Description

The Hospital-Based School Program (HBSP) from which the study was conducted is part of a large, free-standing children’s hospital in the Midwestern United States. It is affiliated with an academic health system and medical school. During the study period, the HBSP employed 14 staff: 11 teachers (seven full-time and four part-time teachers); one permanent substitute teacher (i.e., to fill in for teachers on leave, paid time off, etc.); one full time school manager; and one part time instructional assistant (0.75 FTE). HBSP services within the hospital’s ABH unit are offered continually throughout the year (Ormiston et al., 2024). Student academic records are collected and maintained while adhering to federal laws regarding student/patient privacy in educational and medical settings (e.g., Family Educational Rights and Privacy Act [FERPA], 20 U.S.C. § 1232g; 34 CFR Part 99; Health Insurance Portability and Accountability Act [HIPAA] of 1996).

Services provided by the HBSP included consultation, direct instruction, support for student reentry to their school of record post-hospitalization, and educational advocacy services (Ormiston et al., 2024). Students admitted to the hospital remained enrolled at their school of record while inpatient. Caretakers of individuals within the ABH unit could decline services, which excluded the patient from data collection. Upon discharge, support for reentry included consultations with school of record personnel, recommendations for supports such as an IEP or 504 Plan upon discharge as needed, advocacy for patient healthcare needs upon return to school, and consultation related to accommodations/modifications needed for the student’s educational programming based upon the student’s medical needs. Additional support could include the provision of health-care literacy or direct training for school personnel and/or the student’s classmates when permission was granted by the caretaker and school of record.

## Methods

### Data Collection

Patient medical and educational data were collected for the state department of education for grant reporting purposes. Data regarding educational services and other relevant educational information were entered into the RedCAP (Harris et al., 2009; 2019) database by HBSP staff after educational consultations, direct instruction sessions, and upon discharge. Data entered into RedCAP were periodically monitored via random checks by the HBSP manager for quality assurance purposes. The primary author’s Institutional Review Board approved extraction of de-identified data from the database for use with this study, as shared by the HBSP’s manager (and third author on this study), and the study adhered to all procedural safeguards outlined in the Institutional Review Board proposal. For the present study, data were extracted from the RedCAP database for the 2021–22 academic year.

Within the database, patient primary and secondary diagnoses at discharge were collapsed and aligned with the *Diagnostic and Statistical Manual of Mental Disorders* (5^th^ ed. [DSM-5]; American Psychiatric Association, 2013) categories (e.g., a primary diagnosis of Attention-Deficit/Hyperactivity Disorder was coded a neurodevelopmental disorder; a primary diagnosis of adjustment disorder was coded as trauma- and stressor-related disorder; see Supplemental Table 2). The first author initially coded primary and secondary diagnoses in alignment with the DSM-5 categories. All team members reviewed the codes and discussed and resolved discrepancies to establish the final corpus of diagnostic categories used for analysis. Patients with medical complexity were not admitted to this behavioral health unit per admission guidelines and lack of medical care available within the unit.

### Data Analysis

Analysis was completed with SPSS Version 29 (IBM Corp., 2023) for descriptive and inferential statistics. Chi-square analyses were utilized for comparing across categorical variables while non-parametric tests were utilized for numerical analyses due to heterogeneity of variance amongst groups. Mann-Whitney U tests were used to compare two groups while Kruskal-Wallis Analyses of Variance (ANOVA) were used for three or more groups. Individual odds ratios were also run using MedCalc’s Odds Ratio calculator (MedCalc Software Ltd., 2025) to examine comparability between groups.

## Results

### RQ1: Demographic Characteristics of the Patient Population in the Behavioral Health Unit

Results indicate that the patient population of the behavioral health unit includes patients (n = 264) ranging in age from eight to 17 years old (x̅ = 14.04, SD = 1.89). Of the sample for this study, 70.1% of patients identified as White, 22.0% identified as Black, 6.4% identified as Other (e.g., documented as “unknown” or “refused”), and 1.1% identified as Asian. The majority (72.3%) of the sample identified as female. Half of the patient sample (n = 132, 50.0%) came from the county in which the hospital was located.

The most common diagnosis category was depressive disorders (n = 155; 58.7%). The next highest diagnostic categories were anxiety disorders (n = 27; 14.4%) and neurodevelopmental disorders (n = 25; 9.5%). The average length of stay for the unit was 7.10 days (SD = 2.91) and the average length of the overall hospital stay was 8.87 days (SD = 4.61) with a maximum of 27 and 38 days admitted, respectively. The characteristics of patients’ school of record indicate 76.9% of patients (n = 203) attended public school and 82.2% of patients (n = 217) were being educated in a regular education classroom when admitted. Just less than 4% of patients (n = 10, 3.8%) had a previously established 504 Plan while 14.68% (n = 37) had a prior IEP (see Table 1).

**Table 1:** Demographic and School Characteristics of Patient Sample (n = 252–264).


CHARACTERISTIC	REPRESENTATION IN SAMPLE % (n)

Average age	M = 14.04 (SD = 1.89; n = 263)

Race	

White	70.1% (185)

Black	22.0% (58)

Asian	1.1% (3)

Other	6.4% (17)

Gender	

Female	72.3% (191)

Male	27.3% (72)

Diagnosis Category	

Anxiety Disorders	14.4% (38)

Bipolar & Related Disorders	1.5% (4)

Depressive Disorders	58.7% (155)

Disruptive, Impulse-Control and Conduct Disorders	1.9% (5)

Feeding and Eating Disorders	1.9% (5)

Neurodevelopmental Disorders	9.5% (25)

Other Mental Disorders	2.3% (6)

Other Psychosis	0.8% (2)

Personality Disorders	0.8% (2)

Trauma- and Stressor-Related Disorders	7.6% (20)

Length of Stay	

Average for the unit	M = 7.10 (SD = 2.91; n = 264)

Average for the hospital	M = 8.87 (SD = 4.61; n = 261)

Regularly Attended School of Record	

Alternative	1.5% (4)

Charter	6.1% (16)

Homeschool	2.3% (6)

Private	3.0% (8)

Public	76.9% (203)

Virtual Only	5.7% (15)

Regularly Attended Classroom	

Medical Homebound	0.8% (2)

Outside School Placement	3.4% (9)

Regular Education Classroom	82.2% (217)

Resource Classroom	8.3% (22)

Self-contained Classroom	0.8% (2)

Supports Prior to Inpatient Admission	

Individualized Education Plan (IEP)	14.68% (37)

504 Plan	3.8% (10)


*Note*. sample size varies based on data availability.

### RQ2: School Program Services Offered in the Behavioral Health Unit

Patients received an average of three school consultations (x̅ = 2.96, SD = 1.92) with a range from zero to thirteen. Direct instructions were provided between zero and fourteen times with an average of 4.45 per patient (SD = 2.00). Services after discharge were rarer with patients receiving between zero and two services after discharge (x̅ = 0.52, SD = 0.61). Further analyses revealed there were no differences in the number of services provided by age, sex, or race. There were statistically significant differences in the number of school consultations (dependent variable) by the type of school attended (independent variable). Students from non-public schools were more likely to participate in 0–2 school consultations while students from public schools were more likely to receive three or more school consultations (χ^2^ = 36.252, p = <0.001, Cramer’s V = 0.371). No statistically significant differences were found based on the type of classroom in which the student was taught.

### RQ3: School Program Services Offered in the Behavioral Health Unit by Diagnosis Type

There were no significant correlations between the number of services provided by the HBSP on the behavioral health unit and diagnosis. However, there were some statistically significant relationships between diagnoses and 504 Plan/IEP status at time of admission. Chi-square results indicated no significant differences in prior 504 Plan status at admission, but did find a statistically significant relationship between diagnosis and prior IEP upon admission (χ^2^ = 34.788, p = 0.041). Individuals with neurodevelopmental disorders, psychosis, and “other” mental disorders were found to be most likely to have an IEP prior to admission.

Chi-square results showed a statistically significant relationship between diagnosis and recommendation for a 504 Plan upon discharge (χ^2^ = 36.167, p = 0.029). At discharge, individuals with depressive disorders and anxiety disorders had particularly high rates for receiving a recommendation for a 504 Plan to support reintegration. Conversely, chi-square results found no significant differences in recommendations for an IEP at discharge.

### RQ4: Differences in Behavior Health Unit Service Provision and Diagnosis Based on Demographic Characteristics

Due to heterogeneity of variance within the dataset, an independent-samples Mann-Whitney U-test was used to analyze prior 504 Plan status (dependent variable) in relation to age (independent variable). Age was associated with prior 504 Plan status as older children were more likely to already have one in place at time of admission, (U ([1], N = [252]) = [1803], p = [0.008]). However, there were no statistically significant differences in age related to prior IEP status or in 504 Plan recommendation following interaction with the HBSP on the behavioral health unit. There was a statistically significant difference in IEP recommendation (dependent variable) in relation to age (independent variable). However, in this instance, older children were less likely to receive the recommendation (U ([1], N = [212]) = [761.5], p = [0.012]).

Independent-samples Kruskal-Wallis ANOVA was also used to examine differences in age at time of hospitalization (dependent variable) and the individual’s primary diagnosis (independent variable) (H ([11], N = [263]) = [32.81], p = [< .001]). Differences were also seen between the sex of the patients and their primary diagnosis (χ^2^ = 146.94, p = <0.001). While some diagnoses showed relatively even distributions between males and females (e.g., neurodevelopmental disorders), several others, including anxiety, depression, bipolar, feeding and eating, disruptive, impulse-control, and conduct disorders were much more heavily skewed towards females in this sample. Additional odds ratios were run with the four most common diagnoses (i.e., anxiety, depression, neurodevelopmental disorders, and trauma/stress disorders) to further elucidate these trends (see Table 2). These analyses found no statistically significant differences between males and females as all confidence intervals included 1 (i.e., equality).

**Table 2:** Odds Ratios of Diagnosis by Sex.


PRIMARY CONCERN	FEMALE RATIO	MALE RATIO	OR	95% CI

Anxiety	30 patients with/161 patients without (15.7%)	8 patients with/64 patients without (11.1%)	1.4907	0.6487–3.4253

Depression	116 with/75 without (60.7%)	39 with/33 without (54.2%)	1.3087	.75–2.2616

Neurodevelopmental	14 with/177 without (7.3%)	11 with/61 without (18%)	0.4386	0.1891–1.0177

Trauma/Stress	15 with/176 without (7.8%)	5 with/67 without (6.9%)	1.1420	0.3994–3.2653


*Note*. OR = odds ratio; CI = confidence interval.

When examining race discrepancies in primary diagnoses, a statistically significant difference was found (χ^2^ = 167.92, p = <0.001). However, odds ratios run between the two largest groups (Black students and White students) and focusing on the diagnoses with the largest samples (i.e., anxiety, depression, neurodevelopmental disorders, and trauma/stress disorders) demonstrated minimal differences and confidence intervals that all included 1 (i.e., equality). These results can be seen in Table 3. To further examine these results, odds ratios were also run comparing the ratios of Black patients with particular diagnoses to the ratios of Black individuals in the general population of the state. These data are presented in Table 4 and suggest Black students are over-represented with the diagnoses of depression, neurodevelopmental disorders, and trauma/stress disorders.

**Table 3:** Odds Ratios of Diagnosis by Race.


PRIMARY CONCERN	BLACK	WHITE	OR	CI

Anxiety	5 with/53 without (8.6%)	32 with/153 without (17.3%)	0.4511	0.1671–1.2175

Depression	31 with/27 without (53.4%)	113 with/72 without (61.1%)	0.7316	0.4037–1.3258

Neurodevelopmental	8 with/50 without (13.8%)	14 with/171 without (7.6%)	1.9543	0.7757–4.9238

Trauma/Stress	7 with/51 without (12.1%)	13 with/172 without (7.0%)	1.8160	0.6880–4.7931


*Note*. OR = odds ratio; CI = confidence interval.

**Table 4:** Odds Ratios of Diagnosis by Race Compared to Race Prevalence in the Local Population.


PRIMARY CONCERN	BLACKS IN BHU (n)	BLACKS IN STATE POPULATION (n)	OR	CI

Anxiety	5 Black/32 Others (13.6%)	10 Black/90 Others	1.4063	0.4467–4.4272

Depression	31 Black/124 Others (20%)	10/90	2.25	1.0494–4.8242

Neurodevelopmental	8 Black/17 Others (32%)	10/90	4.2353	1.4608–12.279

Trauma/Stress	7 Blacks/13 Others (35%)	10/90	4.8462	1.5695–14.9639


*Note*. BHU = behavioral health unit; OR = odds ratio; CI = confidence interval.

## Discussion

Behavioral health unit hospitalizations for youth are exceptionally costly (Zima et al., 2016) and have increased significantly in recent years (Arakelyan et al., 2023). The behavioral health and educational needs of youth both while hospitalized as well as post-discharge are complex (Marraccini et al., 2019) and further work is needed to better understand how to support youth during that transition (Midura et al., 2023). Although schools play an important role in supporting youth mental health (Duong et al., 2021), “some youth continue to struggle with the demands [of] the school context and with managing psychiatric symptoms, and some of these youth may be avoiding school which may be hindering their access to school-based mental health programs” (Ogilvie et al., 2019, p. 425).

### Demographics of Patient Population

Most students hospitalized in this behavioral health unit had depressive disorders as their primary diagnosis, consistent with historical trends (Zima et al., 2016) of depressive disorders as the primary diagnosis associated with inpatient hospitalizations (Bardach et al. 2014) and increasing diagnosis of mood disorders (Meagher et al., 2013). Further, most individuals hospitalized in our study (72.3%) were females, consistent with current national trends (Arakelyan et al., 2023; Zima et al., 2016). Interestingly, hospitalization rates for female patients have increased over time while males have decreased (Arakelyan et al., 2023) so perhaps this result is not surprising given the current hospitalization trends. Results of chi-square analyses examining differences related to sex of the patient and their primary diagnosis found some diagnoses typically more prevalent in males (e.g., behavior/conduct disorders; Sappenfield et al., 2024), were more prevalent amongst females in this sample. However, caution should be exercised when interpreting these results as the statistically significant difference seen in the chi-square analysis may be largely driven by diagnoses with particularly small sample sizes, thus warranting further research in this area.

Finally, it is interesting to note the racial differences in diagnoses of the patient population. Black patients in this sample were more likely to be diagnosed with depression, neurodevelopmental disorders (e.g., ADHD), and trauma disorders. This is in alignment with higher incidence rates of trauma exposure and diagnoses of trauma-related disorders (Pumariega et al., 2022) alongside concurrent and prospective associations between trauma exposure and ADHD diagnoses (i.e., associations between early child maltreatment and later ADHD diagnoses; González et al., 2019) in minoritized populations. Within a broader societal context, this finding is noteworthy when considering social determinants of mental health and the outcomes associated with mental health diagnoses. Social determinants of mental health refer to malleable social, cultural, economic, and environmental factors that contribute to the development of mental health concerns and access to related services (Alegría et al., 2018). Social determinants are often operationalized by demographic variables, including economic, language, disability, race, ethnicity, sex, gender, and sexual orientation statuses. Although an in-depth discussion is beyond the scope of this paper, the present study’s results contribute to this literature by considering equitable access to mental health supports for minoritized populations. Specifically, racially and ethnically minoritized youth are less likely to receive outpatient treatment (Bardach et al., 2014), potentially leading untreated symptoms to increase in severity and daily functioning to become so severely impacted that hospitalization is required. While this study did not examine potential interactions by patient sex alongside race and ethnicity due to the sample size, it is possible this interaction may be an underlying interpretation of this study’s data considering the high and increasing rates of depression among females and Black youth (Daly, 2022). Considering this possibility within the present sample’s prevalence rates of depressive disorders and female-identified patients, future work in this area is warranted.

### Educational Services Provided for Youth

Results of the current study indicated students who had a 504 Plan at admission had a higher average age (15.5; n = 10) than those who did not (13.9; n = 242) while students who were recommended an IEP at discharge had a lower average age (12.5; n = 13) than those who were not (14.1; n = 199). Individuals with neurodevelopmental disorders, psychosis, and other mental disorders were found to be most likely to have an IEP prior to admission. At discharge, individuals with depressive disorders and anxiety disorders had particularly high rates to receive a recommendation for a 504 Plan to support reintegration. It was standard practice of the HBSP team to recommend an initial 504 Plan at discharge for patients with no prior supports. This is not unlike the historical trend that individuals with mental health issues are less likely to be identified as having a disability due to mental health needs and thus less likely to receive special education services (Skaar et al., 2021; Yell et al., 2018). Further, 504 Plan eligibility has broader options and requires less intensive needs to be demonstrated compared to obtaining IEP eligibility (Zirkel & Weathers, 2016). There has been a recent increase in students receiving 504 Plans, though as of 2020 approximately 2% of students nationally had a 504 Plan in place (Goodman-Scott & Boulden, 2020) with individuals significantly more likely to be White and male (Zirkel & Weathers, 2016). This is somewhat consistent with our study as most students admitted to the ABH unit were White, although female instead of male.

### Implications for Practice

The importance of mental health care for youth post-ABH unit hospitalization cannot be overstated, considering the challenges youth face upon reintegration in the school setting (Marraccini et al., 2022; Preyde et al., 2017). “Finding ways to enhance collaboration between acute psychiatric hospitals and schools is necessary for students to successfully reintegrate” (Midura et al., 2023, p. 24). While mental health services within the school setting can take place in a variety of ways (i.e., IEPs, 504 Plans), and should not serve as a replacement for comprehensive mental health services (Hoover & Bostic, 2021), the coordination of the services between schools and hospitals is needed.

#### Care Coordination

The coordination of care between medical providers and schools is of utmost importance when considering the care of youth with significant behavioral health needs (Nygaard, Renshaw, et al., 2024). However, the delivery of these care coordination services is informal, inconsistent (Marraccini et al., 2019), and “patchwork” (Nygaard, Ormiston, et al., 2024, p. 1) at best while non-existent at worst (Marraccini et al., 2019; Vanderberg et al., 2023). For instance, only 16% of school psychologists surveyed in a national sample reported their schools as having a formal reintegration plan in place for youth returning from behavioral health unit while 45% reported an informal procedure and 38% reported not having any reentry protocol (Marraccini et al., 2019). Yet, a recent Delphi study conducted with 56 experts across 18 countries over the course of two years resulted in several recommendations for successful reentry of youth post-discharge (Capurso et al., 2025). More specifically, while the recommendations are distinguished between physical health conditions and mental health conditions, common elements emerged across both models, including the presence of a supportive environment, psychological support alongside psychoeducation, the involvement of health coordinators alongside a multidisciplinary team, tailored plan development and monitoring, and collaborative communication between the multidisciplinary team members (Capurso et al., 2025). Further, in alignment with existing literature in the field (e.g., Tougas et al., 2023; UN Convention on the Rights of the Child, 1989), the models’ recommendations center youth within the reentry process and indicate reentry planning should begin at hospital admission rather than at discharge (Capurso et al., 2025).

While the HBSP in the current study engaged in similar consultative practices related to school-hospital care coordination upon reentry, these practices were more commonly present for students attending public schools compared to those in private schools. For youth with significant behavioral health needs, the care coordination practices outlined by Capurso and colleagues (2025) can serve an important link between the hospital, family, and school upon a student’s discharge, as well as a foundation for reintegration plans recommended for youth (Capurso et al., 2025; Marraccini et al., 2019; Nygaard, Renshaw et al., 2024). Thus, additional work is needed to explore the differences in the HBSP’s services offered to youth in the current study.

#### Role of Schools in Supporting Youth with Behavioral Health Needs

The provision of mental health services in schools are typically from school mental health providers (e.g., school counselors, social workers, and school psychologists; Skaar et al., 2021) although community mental health providers (CMHPs) may also provide services within schools (Zabek et al., 2023). Certainly, collaboration with CMHPs, both within and outside of school, is important to support youth mental health upon reintegration (Marraccini et al., 2019) and may provide a critical access point for service delivery to extend the limited services schools can provide (Villarreal & Castro-Villarreal, 2016) since there is a severe shortage of school mental health professionals (Zabek et al., 2023). For instance, although the National Association of School Psychologists (NASP) recommends a ratio of one school psychologist for every 500 students, the current ratio is much higher at a rate of approximately 1:1100. In some states, the ratio is over 1:5,000 (NASP, 2021). Further, when school psychologists are present in schools, their work predominantly focuses on evaluation for special education eligibility (Farmer et al., 2021) despite the robust training school psychologists receive to provide mental and behavioral health services (NASP, 2020). Although most of their time is engaged in evaluation, it seems school psychologists may not be evaluating for or identifying a need for mental health services (Ennis et al., 2017; Etscheidt et al., 2024; Skaar et al., 2021; Yell et al., 2018). For decades, advocates have documented the underutilization and importance of mental health service provision through IEPs (e.g., Schacht & Hanson, 1999). While a diagnosis does not automatically indicate a disability is present (Schacht & Hanson, 1999), the student does need to demonstrate impairment to a marked degree over a period of time (IDEA, 2004). Arguably, for individuals whose behavioral health needs warrant ABH unit hospitalization, it is possible/likely that their behavioral health needs may have impacted their functioning over time. Given the strong, inverse relationship between long term academic outcomes and mental health (Agnafors et al., 2021), it is likely more support is needed for this population. However, in the present study, very few students had an IEP (n = 37; 14.8%) or 504 Plan (n = 10; 3.8%) upon inpatient admission. This was surprising given that mental health services within the school setting can serve an important part of a comprehensive plan to support students with significant mental health needs (Hoover & Bostic, 2021).

Additionally, schools can play a role in the social well-being of youth as they transition from inpatient hospitalization back into the school setting. For instance, planning should be focused on school belonging and school connectedness, as both constructs have been implicated as protective factors for youth with mental health needs and is associated with on time school completion (Kirkpatrick, 2020; Marraccini, Resnikoff, et al., 2022). Coinciding with this, peer social relationships are important to maintain for hospitalized youth as “research has established that social support is a protective factor for patients’ psycho-physical well-being” (Tomberli & Ciucci, 2021, p. 122). Employing a strengths-based approach to social (Midura et al., 2023) and peer support amidst re-integration is an important element in supporting youth. For instance, Youth Peer Support Services, in which young adult Youth Peer Support Workers with current or previous mental health needs provide support to youth with current mental health needs, have shown promising results to support the recovery of individuals with mental health challenges including increases in hope, self-esteem, and treatment engagement (Gopalan et al., 2017). While research with adults in this realm is promising, more work is needed for youth mental health service provision (de Beer et al., 2024). Additionally, there is some evidence to indicate that employing technological interventions (e.g., video chats) with hospitalized youth and their classmates in order to address sense of belonging may be one aspect of support that can help maintain a social connection with peers during their absence due to hospitalization, although active engagement of hospitalized youth is an important element to consider when using these types of interventions (Tomberli & Ciucci, 2021). In sum, while there is some evidence to indicate schools can play a role in providing adult mentoring and peer-oriented supports to hospitalized youth (Marraccini, Pittleman, et al., 2022), additional work in this area is needed.

### Limitations and Directions for Future Research

Several limitations must be acknowledged for the current study. First, the demographics of the sample represented a relatively racially/ethnically homogenous sample thus limiting the ability to conduct some analyses. For instance, the sample sizes for Black patients with neurodevelopmental disorders and trauma/stress disorders are not particularly robust (n = 8 and n = 7, respectively). Thus, future research with more diverse and representative samples is needed. Additionally, the youth in the present study all presented with psychiatric diagnoses and medical needs were exclusionary to ABH admission at the site where the study took place (i.e., youth with medical and behavioral health needs receive medical care in other units with psychiatric consult and liaison services demonstrating a fundamentally different experience from ABH admission). Thus, additional research should examine trends related to the presence of medical diagnosis and examine the educational services of youth with comorbid medical and psychiatric diagnoses. Although we examined educational services provided to students in the ABH as well as recommendations for educational services via an IEP or 504 Plan post-discharge, we did not have the ability to determine if schools followed through on those recommendations, nor do we have information relating to students’ academic functioning upon return to their home schools. Further, because we did not measure academic functioning while inpatient or upon return to their home school, we do not know if the services provided by the ABH were of benefit to the student. Finally, we did not examine the patient population to determine what additional school or community based supports the individuals may have had, if any. Future research should examine whether school-based mental health services, as part of comprehensive coordinate care efforts, can help prevent the return to the ABH. Taken together, while the present study’s results add to the existing literature base, these limitations must be considered when applying interpretation of the results and implications for practice.

## Conclusion

The behavioral health needs of youth returning to school post-discharge from a behavior health stay should not be minimized. Youth returning to their school of record should be provided with comprehensive reintegration plans to support their transition (Marraccini et al., 2019). Although the demographics of youth in the current study of patients in a Midwestern behavioral health unit mirror national trends (e.g., Zima et al., 2016), this study provided some detail as to the types of educational services provided to youth prior to, during, and post-inpatient hospitalization. Specifically, few patients received any type of formal school-based support, either through a 504 plan or an IEP, prior to admission to the ABH unit. Differences were found between service utilization based on the type of school attended, and certain diagnoses (e.g., depression, anxiety) were more likely to be recommended for in-school supports post-discharge. Additional research is needed to better understand the educational and behavioral health needs of this population as they return to their school of record to maximize behavioral health over time. Specifically, further examining the longitudinal impact of inpatient and post-discharge services and recommendations in terms of post-discharge functioning and re-admission rates would significantly add to the literature base of this population. Additionally, further examining the impact of school-based mental health supports and the relationship to post-discharge and re-admission rates is a much-needed area of study.

## Additional File

The additional file for this article can be found as follows:

10.5334/cie.178.s1 Supplementary Material. Tables 1 and 2.
