# Supplementary material for: Educational Services and School Reintegration Supports for Youth After Acute Behavioral Health Unit Hospitalization
Source: Contin Educ. 2025 Oct 29;6(1):162–78. doi: 10.5334/cie.178 (PMC12577547; doi:10.5334/cie.178)
Supplement: Supplementary Material. — Tables 1 and 2. [file cie-6-1-178-s1.zip › cie-178_ormiston-s1/Supplemental Table 1.pdf]

Educational Services and School Reintegration Supports for Youth After Acute Behavioral Health Unit Hospitalization

Heather E. Ormiston, Polly R. Husmann, Kristen Wikel, Debra L. Reisinger, Michelle Curtin

Supplemental Table 1

Coded Patient Primary and Secondary Diagnoses

Coded Diagnosis	Examples of Disorders
Neurodevelopmental Disorders	Autism spectrum disorder
	Attention-deficit/hyperactivity disorder (ADHD)
	Learning disorders (covers dyslexia, dyscalculia, etc.)
Schizophrenia Spectrum and Other Psychotic Disorders	Schizophrenia
	Schizoaffective disorder
	Delusional disorder
Bipolar and Related Disorders	Bipolar I and bipolar II disorders
	Cyclothymic disorder
Depressive Disorders	Major depressive disorder
	Persistent depressive disorder
Anxiety Disorders	Generalized anxiety disorder
	Social anxiety disorder
	Separation anxiety disorder
	Panic disorder
	Phobias
Obsessive-Compulsive and Related Disorders	Obsessive-compulsive disorder (OCD)
	Hoarding disorder
	Body dysmorphic disorder
	Skin-picking disorder and hair-pulling disorder
Trauma- and Stressor-Related Disorders	Post-traumatic stress disorder (PTSD)
	Acute stress disorder
	Adjustment disorder

This document contains supplementary material for the above-mentioned article, as provided by the authors.

The original article can be downloaded from https://doi.org/10.5334/cie.178

Dissociative Disorders	Dissociative identity disorder
	Dissociative amnesia
	Depersonalization/derealization disorder
Somatic Symptom and Related Disorders	Somatic symptom disorder
	Illness anxiety disorder
	Functional neurological symptom disorder (conversion disorder)
Feeding and Eating Disorders	Anorexia nervosa
	Bulimia nervosa
	Binge-eating disorder
	Pica
Elimination Disorders	Enuresis
Sleep-Wake Disorders	Insomnia disorder
	Narcolepsy
	Sleep apnea disorders
	Nightmare disorder
	Restless legs syndrome
Sexual Dysfunctions	Sexual dysfunctions
Gender Dysphoria	Gender dysphoria-related disorders
Disruptive, Impulse-Control and Conduct Disorders	Oppositional defiant disorder
	Antisocial personality disorder
	Kleptomania
	Pyromania
Substance-Related and Addictive Disorders	Alcohol use disorder
	Inhalant use disorder
	Opioid use disorder
	Withdrawal-related symptoms
Neurocognitive Disorders	Delirium
	Alzheimer’s disease
	Parkinson’s disease
	Huntington’s disease

This document contains supplementary material for the above-mentioned article, as provided by the authors.
The original article can be downloaded from https://doi.org/10.5334/cie.178

	Traumatic brain injury
Personality Disorders	Borderline personality disorder (BPD)
	Narcissistic personality disorder
Paraphilic Disorders	Sexual behavior disorders
Other Mental Disorders and Additional Codes	Conditions that don't match the definition of another condition, but that still significantly affect someone's life
Medication-Induced Movement Disorders and Other Adverse Effects of Medication	Tardive dyskinesia<br>Neuroleptic malignant syndrome

This document contains supplementary material for the above-mentioned article, as provided by the authors.
The original article can be downloaded from https://doi.org/10.5334/cie.178
