# Supplementary material for: Educational Services and School Reintegration Supports for Youth After Acute Behavioral Health Unit Hospitalization
Source: Contin Educ. 2025 Oct 29;6(1):162–78. doi: 10.5334/cie.178 (PMC12577547; doi:10.5334/cie.178)
Supplement: Supplementary Material. — Tables 1 and 2. [file cie-6-1-178-s1.zip › cie-178_ormiston-s1/Supplemental Table 2.pdf]

Educational Services and School Reintegration Supports for Youth After Acute Behavioral Health Unit Hospitalization

Heather E. Ormiston, Polly R. Husmann, Kristen Wikel, Debra L. Reisinger, Michelle Curtin

Supplemental Table 2

US Specific Legal References for the Provision of Health- and Education-Related Services

US Specific Legal References	Definition and Provisions
Health Insurance Portability and Accountability Act (HIPAA)	US Department of Health and Human Services federal law protecting a patient's private and secure health information<br>Protected health information cannot be shared without patient/parent/legal guardian's permission<br>Law applies to healthcare entities (e.g., clinics, hospitals), healthcare providers (e.g., doctors, therapists), and business that act on behalf of the covered entity (e.g., insurance companies)
Family Education Rights and Privacy Act (FERPA)	US Department of Education federal law protecting a patient's/student's academic records<br>Applies to any educational institution that receives federal funds<br>Personally identifiable information cannot be shared without parent/guardian/student's permission unless student is 18 years old or enrolled in college<br>Applies to any public, private, state, or local education agency
Individualized Education Plan (IEP)	US Department of Education Office of Special Education Programs Individuals with Disabilities Education Act<br>Federal funding<br>Disability requires special education and specialized instruction beyond what is typically provided in the general education classroom<br>Addresses needs of individuals from birth up to 21 years of age
Section 504 Pan	US Department of Education Office of Civil Rights provision of services or individuals with a disability that do not meet eligibility criteria for special education services<br>A student must have a physical or mental impairment that significantly limits one, or more, major life activities<br>"Meant to prevent discriminatory practices against children with disabilities in public schools, but federal funding is not provided for this explicit purpose" (NASP, 2023, p. 1)<br>Disability does not require individualized instruction beyond the general education classroom<br>Addresses needs of individuals across the lifespan

This document contains supplementary material for the above-mentioned article, as provided by the authors.

The original article can be downloaded from https://doi.org/10.5334/cie.178

(Centers for Disease Control and Prevention, 2024; National Association of School Psychologists [NASP], 2023; Zirkel, 2015)

This document contains supplementary material for the above-mentioned article, as provided by the authors.

The original article can be downloaded from https://doi.org/10.5334/cie.178
